# Habitat Model Based on Lung CT for Predicting Brain Metastasis in Patients with Non-Small Cell Lung Cancer

**DOI:** 10.3390/diagnostics15233043

**Published:** 2025-11-28

**Authors:** Feiyu Xing, Yan Lei, Qin Zhong, Yan Wu, Huan Liu, Yuanliang Xie

**Affiliations:** 1 School of Medicine, Jianghan University, Wuhan 430056, China; xingfeiyu0717@gmail.com (F.X.); foryou7913@163.com (Y.L.); 2 Department of Radiology, The Central Hospital of Wuhan, Tongji Medical College, Huazhong University of Science and Technology, Wuhan 430014, China; zqwh2023@163.com (Q.Z.); juliayanzi@163.com (Y.W.); 3 GE Healthcare, Advanced Analytics Team, Shanghai 201203, China; huan.liu@gehealthcare.com; 4 Department of Radiology, Yichang Central People’s Hospital, No. 183 Yiling Avenue, Yichang 443000, China

**Keywords:** lung cancer, brain metastasis, radiomics, habitat

## Abstract

**Background**: In lung cancer, the occurrence of brain metastasis (BM) is closely associated with the heterogeneity of the primary lung tumor. This study aimed to develop a habitat-based radiomics model using enhanced computed tomography (CT) lung imaging to predict the risk of BM in patients with non-small cell lung cancer (NSCLC). **Methods**: A retrospective cohort of 475 patients with NSCLC who underwent enhanced CT of the lungs prior to anti-tumor treatment was analyzed. Volumetric CT images were segmented into tumor subregions via k-means clustering based on voxel intensity and entropy values. Radiomics features were extracted from these subregions, and predictive features were selected using minimum redundancy maximum relevance and least absolute shrinkage and selection operator regression. Two logistic regression models were constructed: a whole-tumor radiomics model and a habitat-based model integrating subregional heterogeneity. Model performance was evaluated via receiver operating characteristic analysis and compared via DeLong’s test. **Results**: A total of 195 eligible patients with NSCLC were included. The volume of interest of the whole tumor was clustered into three subregions based on voxel intensity and entropy values. In the training cohort (*n* = 138), the areas under the curve of the clinical model, the whole-tumor model and the habitat-based model were 0.639 (95% confidence interval [CI]: 0.543–0.731), the whole-tumor model and the habitat-based model were 0.728 (95% confidence interval [CI]: 0.645–0.812) and 0.819 (95% CI: 0.744–0.894), respectively. The habitat-based model demonstrated superior predictive performance compared with the whole-tumor model (*p* = 0.022). **Conclusions**: The habitat-based radiomics model outperformed the whole-tumor model in terms of predicting BM, highlighting the importance of subregional tumor heterogeneity analysis.

## 1. Introduction

Brain metastasis (BM) is a devastating complication of non-small cell lung cancer (NSCLC), occurring in approximately 10–30% of patients during disease progression and correlating with severe neurological morbidity and a median survival of less than 12 months despite treatment advances [1,2]. Early prediction remains a critical unmet clinical need; conventional surveillance strategies, such as routine magnetic resonance imaging (MRI) or symptom-based diagnosis, often fail to detect micrometastases at treatable stages, while prophylactic interventions such as cranial irradiation carry significant risks of neurocognitive decline [3,4]. Certain indicators of tumor nature such as gene mutation status and tumor stage have been used as predictive biomarkers to predict BM, as have several conventional imaging signs, including tumor size, location, and enhancement. The discriminatory power, however, remains limited, with areas under the curve (AUCs) of <0.75 in most studies, underscoring an urgent demand for more precise non-invasive predictive tools. Generally, early-stage lung cancer is detected by low-dose computed tomography (CT) of the lungs, and brain imaging is not routinely performed, which means that the risk of BM may be underestimated [5].

Although a combination of multimodal imaging methods, such as CT, MRI, and nuclear medicine, is commonly used to detect BM in patients with lung cancer, the efficiency and sensitivity are still unsatisfactory, and the final diagnosis may be delayed, affecting individual treatment decisions [6]. Lung CT plays an important role in NSCLC diagnosis and tumor stage assessment; however, there are no sensitive applicable imaging markers for predicting BM. Therefore, it is necessary to propose a new, non-invasive method for screening patients with NSCLC at high risk for BM.

Radiomics has emerged as a transformative approach in oncology, leveraging high-throughput extraction of quantitative features from medical images to decode tumor heterogeneity and microenvironmental dynamics invisible to the human eye [7]. By translating pixel-level data into minable biomarkers, radiomics has demonstrated superior performance in predicting tumor behavior, including grading, lymph node metastasis, and treatment response [8,9]. A few studies have explored the potential value of radiomics analyses for BM prediction in patients with NSCLC [10]. However, traditional radiomics approaches often treat lung tumors as homogeneous entities, overlooking their spatial and functional heterogeneity, which may drive metastatic aggressiveness. This limitation has spurred an interest in habitat imaging analysis, an advanced radiomics paradigm that partitions tumors into subregions based on distinct radiologic or pathophysiological signatures, thereby mapping the ecological interplay between tumor subclones and their microenvironment [11].

Early studies suggest that habitat-driven radiomics features, such as edge-enhancing gradient variations and perfusion heterogeneity, correlate with invasive phenotypes and metastatic potential [12,13]. Yet, the potential value of habitat imaging as a tool for predicting NSCLC BM remains underexplored, with no validated models integrating spatially resolved biomarkers into clinical prognostication.

In this study, we developed a habitat radiomics model based on pretreatment lung CT for predicting BM risk in patients with NSCLC. By integrating subregion-specific texture, shape, and wavelet features, we aimed to identify habitat signatures associated with metastatic dissemination to the brain and develop a machine learning framework capable of stratifying patients by their risk of BM at initial diagnosis.

## 2. Materials and Methods

### 2.1. Patient Population

A total of 475 patients with NSCLC who underwent enhanced CT of the lungs before treatment on admission from January 2018 to December 2021 in the Central Hospital of Wuhan (Nanjinglu Branch, Houhu Branch, and Yangchunhu Branch) were included. Inclusion criteria were (1) diagnosis of NSCLC confirmed by pathology, including percutaneous puncture biopsy, endoscopic biopsy, and postoperative pathology; (2) pretreatment enhanced chest CT performed within 1 month prior to surgery or biopsy; (3) availability of complete clinical variables and records of 3-year follow-up; (4) availability of test results for at least 10 genes, namely STK11, EGFR, SETBP1, TP53, ALK, FAT1, KRAS, MET, ROS1, and HER2; (5) availability of qualified CT images of the aortic arterial phase in which the aorta measured ≥300 Hounsfield units (HU); (6) T stage ≥ Ib; and (7) treatment following the Expert Consensus in Lung Cancer Treatment (China 2020 Version) guidelines. Exclusion criteria were (1) presence of other tumors in addition to lung cancer, (2) prior treatment with antitumor therapy, (3) iodine allergy or other enhanced CT contraindications, (4) presence of severe diseases with an expected survival of <90 days, and (5) poor image quality affecting radiomics analysis. Finally, 195 patients were included. They were divided into a training cohort (*n* = 138) and a validation cohort (*n* = 57) at a ratio of 7:3. Each cohort was further divided into a BM subgroup and a non-BM subgroup. Aortic phase enhanced CT images were used for habitat radiomics analysis.

**Figure 1:**
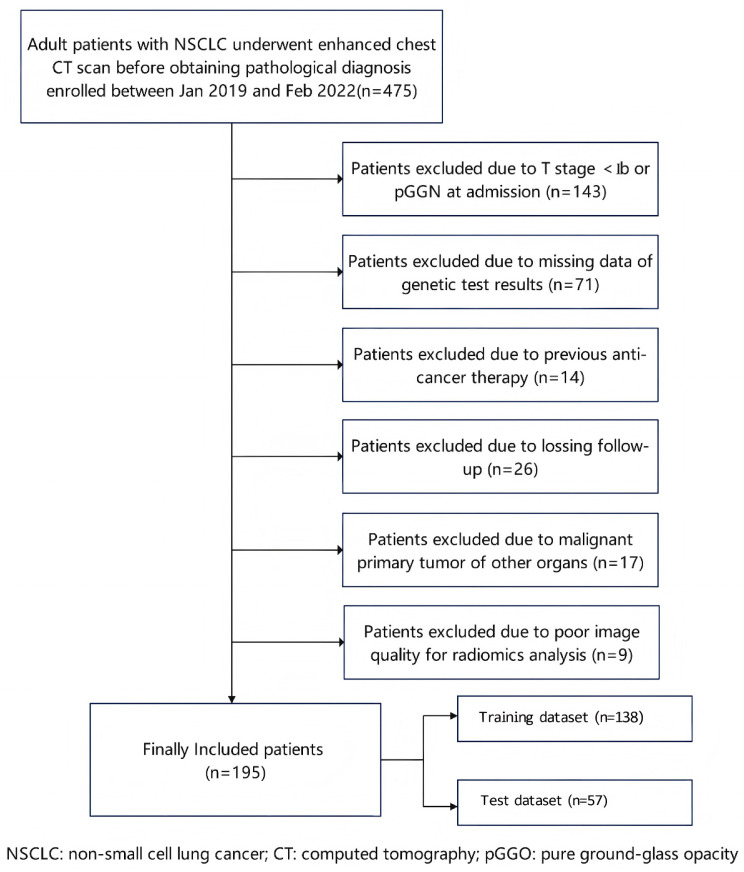
Flow diagram of patient enrollment. NSCLC: non-small cell lung cancer; CT: computed tomography; and pGGN: pure ground-glass nodule.

This retrospective study was approved by the Wuhan Central Hospital Review Committee (WHZXKYL2023-079, 6 July 2023). The requirement for written informed consent was waived due to the study’s retrospective nature. The study protocol adhered to the principles and requirements of the Declaration of Helsinki. Figure 1 shows the flow diagram of patient enrollment.

### 2.2. CT Acquisition and Image Reconstruction

Enhanced chest CT was performed using multi-slice spiral CT scanners (Aquilion ONE, Canon Healthcare; Tokyo, Japan; Somatom Definition AS, Siemens Healthcare, Erlangen, Germany; iCT, Philips Medical Systems, Best, The Netherlands), and arterial phase CT images were used for radiomics analysis. The main parameters included a tube voltage of 120 kVp, tube current of 100–300 mA, detector collimation of 0.5–0.625 mm, field of view of 380 mm, and rotation time of 0.5–0.6 s. Each CT image was routinely reconstructed with a matrix of 512 × 512 pixels and a standard slice thickness of 1 mm using a soft-tissue convolution kernel. A nonionic iodinated contrast medium (Ioversol, 320 mg I/mL) was administered at a dose of 1.5 mL/kg with a flow rate of 3.0 mL/s. We used an iterative reconstruction algorithm to improve the signal-to-noise ratio.

### 2.3. Segmentation and Radiomics Processing

First, all volumetric CT data were uniformly resampled into a voxel resolution size of 1 mm × 1 mm × 1 mm to reduce heterogeneity among images obtained from different CT scanners. Two radiologists (Y, Wu and Q, Zhong), each with 5 years of experience, who were blinded to pathological outcomes, semi-automatically segmented the tumor lesions using ITK-SNAP Ver3.8.0 (www.itksnap.org, accessed on 17 July 2025). A region growing method based on the CT threshold of the tumor was applied and manually corrected when necessary to identify the volume of interest (VOI) and exclude non-tumor tissues, such as vessels, and areas affected by atelectasis. To ensure accuracy and reproducibility, 30 patients were randomly selected within 1 week after the first segmentation. The VOIs were segmented by two readers at time points TP0 and TP1, and radiomics features were extracted accordingly. The intraclass correlation coefficient (ICC) was used to assess inter-observer and intra-observer variability in segmentation and feature extraction. An ICC ≥ 0.70 indicated high feature stability.

**Figure 2:**
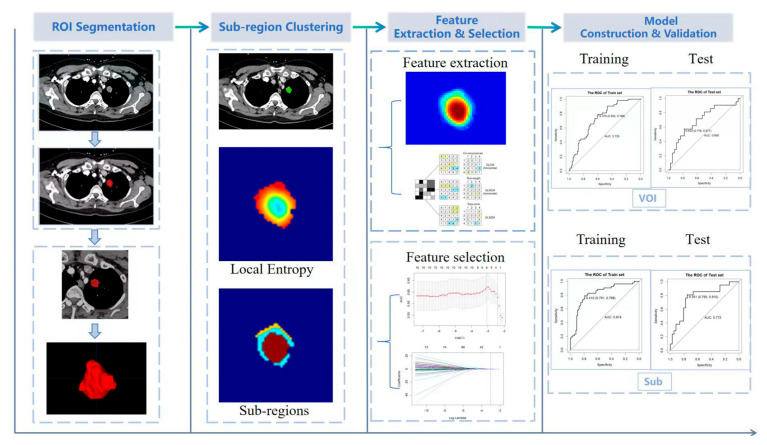
Workflow of radiomics analysis. ROI: region of interest; VOI: volume of interest.

The traditional radiomics process, comprising feature extraction, feature screening and selection, model construction, and predictive performance evaluation, was implemented using the open-source Python package Pyradiomics 1.2.0 (https://pyradiomics.readthedocs.io/en/latest/changes.html#pyradiomics-1-2-0, accessed on 1 September 2025) [14]. A total of 1218 radiomics features were extracted, including first order, shape, gray level co-occurrence matrix, gray level size zone matrix, gray level run length matrix, neighboring gray tone difference matrix, and gray level dependence matrix. Figure 2 depicts the workflow of the radiomics analysis.

### 2.4. VOI Delineation and Subregion Clustering

Habitat analysis was performed using voxel intensity and entropy values derived from CT images to cluster VOIs into subregions [15,16]. The voxel counts for each tumor VOI were determined using a traditional method, and the entropy values for each layer of the CT images were computed using the following formula:

(1) $$V_{voxel}=\sum_{k=1}^{N_{\upsilon }}V_{k}$$

(2) $$Entropy=-\sum_{i=1}^{N_{g}}p\left(i\right)\log_{2}\left[p\left(i\right)+\epsilon \right]$$

The *k*-means method was used to cluster the VOI regions at the population level, forming multiple habitats, and the distance correlation between samples was calculated using the Euclidean distance (voxel intensity values and entropy values).

### 2.5. Feature Selection and Model Development

To reduce the number of redundant radiomics features, subregional radiomics features with a correlation coefficient greater than 0.75 were excluded [17]. We used both the least absolute shrinkage and selection operator (LASSO) and maximum-relevance minimum-redundancy methods to identify features strongly associated with BM and bone metastasis, using a 10-fold cross-validation strategy. A radiomics score was then calculated based on the selected subregional radiomics features and corresponding coefficients. Two prediction models were subsequently developed: one as a Cox proportional hazards model based on the radiomics score, and the other as a radiomics nomogram for visual interpretation [18].

### 2.6. Statistical Analysis

Statistical analyses were performed using SPSS 26.0 (IBM, Armonk, NY, USA) and R 4.3.3 (https://www.r-project.org/, accessed on 30 July 2025; Vienna, Austria). Continuous variables were presented as the mean ± standard deviation (SD) and compared using the Mann–Whitney U test. Categorical variables were expressed as counts with percentages and compared using the χ^2^ test or Fisher’s exact test. The predictive performance of the models was evaluated by constructing a receiver operating characteristic (ROC) curve and calculating specificity, sensitivity, and accuracy. To compare the prediction capabilities of different models, the AUC and Akaike information criterion (AIC) were compared using DeLong’s test. Decision curve analysis was used to assess the clinical applicability of the models. Calibration curves were generated to evaluate the agreement between observed and predicted probabilities.

## 3. Results

### 3.1. Clinical Characteristics

The clinical characteristics of the patients in the training and validation cohorts are shown in Table 1. The mean ages of the patients in the training and validation cohorts were 65.76 ± 9.15 years and 65.95 ± 8.12 years, respectively. Among them, 38 cases were classified as TNM stage I; 61, as stage II; 46, as stage III; and 50, as stage IV. Adenocarcinoma was present in 169 cases, and squamous cell carcinoma was present in 26 cases. There were statistical differences in terms of neuro-specific enolase (NSE) levels and tumor type. Other clinical characteristics such as sex, smoking history, tumor location, gene mutations and levels of tumor markers such as carcinoembryonic antigen (CEA), carbohydrate antigen 125 (CA-125), carbohydrate antigen 72-4 (CA72-4), squamous cell carcinoma antigen (SCC), pro-gastrin releasing peptide (ProGRP), and cytokeratin 19 fragment (Cyfra21-1), did not exhibit statistically significant differences in either group. In addition, the rate of BM occurrence was higher in demographic groups characterized by non-smoking history and male sex. To enhance comparability, we categorized age as <65 or ≥65 years, CEA as >5, CA125 as >35, NSE as >18, serum gastrin-releasing peptide precursor as >70, CK19 as >2.08, and SCC as >2. Multivariate logistic regression analysis identified the following as independent risk factors: age ≥ 65 years, N2 stage, gene mutation, and EGFR mutation. The constructed clinical prediction model yielded an area under the curve (AUC), as well as specificity and sensitivity, as shown in Table 2.

### 3.2. Feature Selection

**Figure 3:**
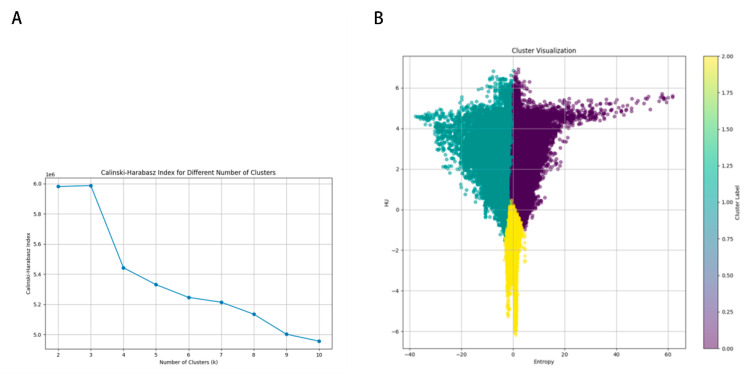
K-means population clustering. (**A**) Calinski–Harabasz index for different numbers of clusters. (**B**) Cluster visualization.

**Figure 4:**
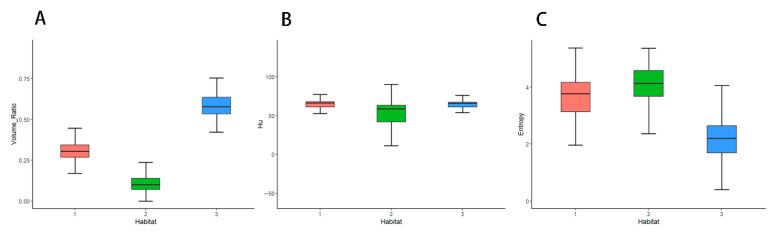
Box-and-whisker plots displaying the distribution of volume ratio (**A**), HU (**B**), and entropy (**C**) on enhanced computed tomography maps.

**Figure 5:**
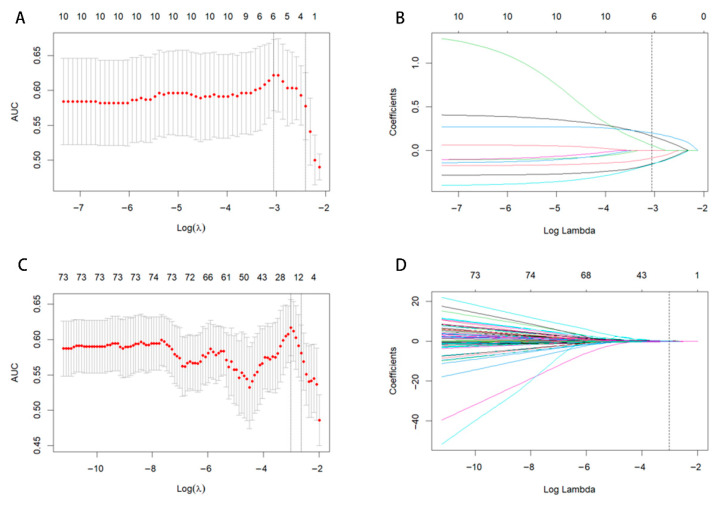
LASSO coefficient path diagram and LASSO cross-verification curve. Optimal whole-tumor radiomics (**A**,**B**) and subregional radiomics (**C**,**D**) were selected.

The optimal Calinski–Harabasz (CH) value in the training cohort was achieved with three clusters. Therefore, the tumor region was divided into three subregions because a statistical difference was present in both the voxel intensity and entropy values, whereas no significant difference in HU values was observed (Figure 3). The optimal k-value was identified as 3. Texture analysis of the structure produced a set of scalar values summarizing the texture of the region (Figure 4) and showing the heterogeneity of the subregion. After redundant radiographic features were eliminated, 1218 conventional radiomics features were extracted from each of the three subregions (totaling 3654 features) and used for further analysis. Based on 5-fold cross-validation, LASSO regression was used to select the optimal subregional radiomics features from the training cohort, and 12 features were ultimately selected for model construction (Figure 5).

### 3.3. Subregional Radiomics Prediction Model

Using the coefficient of each radiomics feature in the LASSO method, radiomics scores of the whole tumor (*VOI*) and subregions (*Sub*) were constructed as follows:

(3) $$\begin{array}{ll} \mathrm{Radscore}(\mathrm{VOI})= & -1314.76350183194\\ & +-0.049373886121726\times \mathrm{original}\_\mathrm{firstorder}\_\mathrm{InterquartileRange}\\ & +-3.3462260807435\\ & \times \log.\mathrm{sigma}.2.0.mm.3D\_\mathrm{glrlm}\_\mathrm{ShortRunLowGrayLevelEmphasis}\\ & +-2.45489742195494\\ & \times \log.\mathrm{sigma}.5.0.\mathrm{mm}.3D\_\mathrm{glszm}\_\mathrm{SizeZoneNonUniformityNormalized}\\ & +37.5565903019046\times \log.\mathrm{sigma}.4.0.\mathrm{mm}.3D\_\mathrm{glcm}\_\mathrm{Idn}\\ & +5112.07298466082\times \mathrm{wavelet}.\mathrm{LHH}\_\mathrm{glrlm}\_\mathrm{GrayLevelVariance}\\ & +0.415506640475669\\ & \times \mathrm{wavelet}.\mathrm{HLH}\_\mathrm{glszm}\_\mathrm{SmallAreaHighGrayLevelEmphasis} \end{array}$$

(4) $$\begin{array}{ll} \mathrm{Radscore}(\mathrm{Sub})= & 49.263094558148\\ & +0.49585946384375\\ & \times \mathrm{Sub}1\_\mathrm{wavelet}.\mathrm{LLH}\_\mathrm{glszm}\_\mathrm{SmallAreaHighGrayLevelEmphasis}\\ & \times -3.11826715000125\times \mathrm{Sub}1\_\log.\mathrm{sigma}.5.0.\mathrm{mm}.3D\_\mathrm{glcm}\_\mathrm{Correlation}\\ & +0.0477379368324887\\ & \times \mathrm{Sub}3\_\log.\mathrm{sigma}.1.0.\mathrm{mm}.3D\_\mathrm{firstorder}\_\mathrm{Kurtosis}\\ & \times -99.5853551879633\\ & \times \mathrm{Sub}2\_\mathrm{wavelet}.\mathrm{LHH}\_\mathrm{glrlm}\_\mathrm{GrayLevelNonUniformityNormalized}\\ & +0.00297377200986883\\ & \times \mathrm{Sub}3\_\mathrm{wavelet}.\mathrm{LLL}\_\mathrm{gldm}\_\mathrm{LargeDependenceLowGrayLevelEmphasis}\\ & +1.21232489590441\times 10^{-8}\\ & \times \mathrm{Sub}3\_\mathrm{wavelet}.\mathrm{LHH}\_\mathrm{glszm}\_\mathrm{LargeAreaLowGrayLevelEmphasis}\\ & +-1.23756693776612\\ & \times \mathrm{Sub}3\_log.\mathrm{sigma}.5.0.mm.3D\_\mathrm{glcm}\_\mathrm{ClusterProminence} \end{array}$$

**Figure 6:**
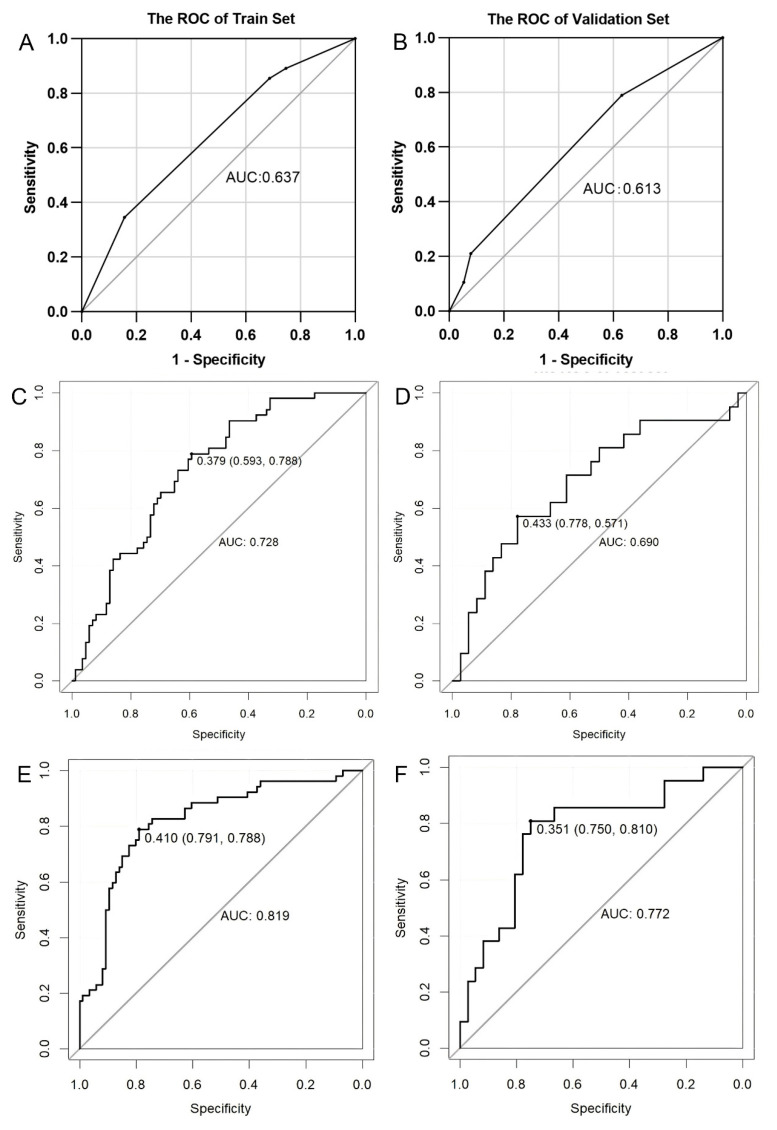
ROC analysis of brain metastasis prediction models. Middling predictive values of the clinical model both in the training (**A**) and validation (**B**) cohorts. The AUCs of the whole-tumor radiomics model in the training (**C**) and validation (**D**) cohorts. The AUCs of the subregional radiomics model in the training (**E**) and validation (**F**) cohorts were higher than those of the whole-tumor radiomics model. AUC: area under the curve; ROC: receiver operating characteristic.

Two BM prediction models were constructed, one based on Radscore_VOI_ and one based on Radscore_Sub_. As shown in Figure 6, the habitat-based subregional model performed well, with AUCs of 0.819 (95% CI, 0.744–0.894) and 0.772 (95% CI, 0.641–0.904) in the training and validation cohorts, respectively. The whole-tumor model produced AUCs of 0.728 (95% CI, 0.645–0.812) and 0.690 (95% CI, 0.542–0.839) in the training and validation cohorts, respectively. The prediction efficiency of the two models is summarized in Table 2. According to DeLong’s test, the habitat-based subregional model performed better than the whole-tumor model (Z = 2.287, *p* < 0.05).

## 4. Discussion

BMs in NSCLC cases are highly variable in terms of occurrence and timing and are influenced by multiple factors. In this study, we used habitat-based radiomics features derived from pretreatment enhanced CT images to predict the risk of BM in patients with NSCLC. The habitat model (AUC: 0.819) had superior predictive performance compared with both the clinical model (AUC: 0.639) and the whole-tumor radiomics model (AUC: 0.728), underscoring the critical importance of spatial heterogeneity in metastasis prognostication. This finding challenges the conventional paradigm of treating tumors as homogeneous entities and provides substantial support for the emerging study on habitat imaging in oncology. The significant performance gain over the clinical baseline confirms that CT-derived habitat features provide unique, incremental prognostic information beyond standard clinical factors.

The subregional heterogeneity captured by our habitat model likely reflects underlying biological processes that drive metastatic progression. The spatially distinct patterns observed on contrast-enhanced CT may correspond to variations in tumor microenvironmental conditions. Specifically, the uneven density and abnormal structure of tumor blood vessels result in an uneven supply of oxygen. Hypoxic regions, which typically appear as poorly enhanced necrotic areas, can promote epithelial–mesenchymal transition and enhance tumor cell invasiveness through hypoxia-inducible factor-1α mediated pathways [19]. Simultaneously, oxygen-rich regions form a significantly enhanced solid area, may indicate active angiogenesis, providing vascular conduits for tumor cell dissemination [20]. The peripheral blood vessels are dense, while the central vessels are sparse, resulting in a “ring enhancement” pattern. This pattern frequently observed in the high-risk cohort may represent an aggressive tumor phenotype with proliferative periphery and necrotic core [21].

Our findings have immediate implications for clinical practice. The habitat risk stratification model enables identification of high-risk NSCLC patients at initial diagnosis, For these high-risk patients, we recommend an intensified surveillance protocol with brain MRI every 3–6 months to enable earlier detection of asymptomatic brain metastases [22]. Additionally, in patients with eligible driver mutations (e.g., EGFR or ALK), our model could support the earlier incorporation of blood–brain barrier penetrating targeted therapies [23]. Conversely, for low-risk patients identified by our model, reduced surveillance frequency could minimize unnecessary radiation exposure and healthcare costs while maintaining oncological safety.

Various researchers have applied habitat imaging to radiomics and produced effective models for the analysis of several types of tumors. Gong et al. [24] developed and validated an online individualized model for predicting local recurrence-free survival in patients with esophageal squamous cell carcinoma treated with definitive chemoradiotherapy. They divided the entire volume of the tumor and surrounding tissue into three subregions by cluster analysis based on CT HU values and local entropy values. The subregional radiomics signature produced better prognostic performance than the whole-tumor-based radiomics signature. Xie et al. [25] investigated the potential of the subregional radiomics signature as a novel tumor biomarker for predicting overall survival in patients with esophageal squamous cell carcinoma treated with concurrent chemoradiotherapy. An overall survival prediction model combining seven subregional radiomics features was constructed, and the prediction efficiency of the subregional model was better than that of the whole-tumor-based model. Chen K et al. [26] studied the prognostic value of habitat radiomics-based CT images in hepatocellular carcinoma and identified habitat radiomics models as the optimal predictive model, with a mean AUC of 0.806. Chen H et al. [27] developed an MRI-based habitat radiomics model to distinguish among different HER2 expression states and quantify the heterogeneity of breast tumors. Yang et al. [28] built a CT-based habitat radiomics signature to evaluate the risk of metastasis in patients with clear cell renal cell carcinoma. These studies demonstrated the value of habitat imaging. Our work significantly extends these habitat study by demonstrating that habitat analysis of the primary tumor can specifically predict the risk of distant brain metastases. Whereas previous research has either relied on conventional whole-tumor radiomics [29], ignoring the habitat of the primary tumor, or focused on the brain metastases themselves to infer the primary tumor [30]. Therefore, we aimed to predict BM risk by analyzing primary lung tumor habitats and stratify patients with NSCLC according to risk upon admission, providing novel insights into thoracic oncology.

The factors influencing BM are complex [31,32,33]. BM is a multi-stage process in which tumor cells are shed from the primary site, invade the surrounding matrix, enter the blood circulation, cross the blood–brain barrier, and colonize and grow in the brain. Lung cancer cells breach the blood–brain barrier through a variety of mechanisms including the secretion of proteases, which degrade tightly connected components. For example, cancer cells overexpressing cyclooxygenase 2 (COX2) and heparin bind to enzymes, such as epidermal growth factor, that help the cells cross the blood–brain barrier. The interaction between tumor cells and the brain microenvironment (including immune cells, astrocytes, microglia, and neurons) is crucial for the formation of BM [34]. Obtaining complete information about all of these factors is time-consuming and expensive, our CT-based habitat model offers a pragmatic and powerful solution. To improve lung cancer treatment decisions, novel methods of stratifying NSCLC BM risk are needed, and high-risk populations must receive brain MRI scans regularly to detect BM earlier. The ability of CT-based habitat features to predict BM underscores that metastatic propensity is encoded in the spatial organization of primary tumors. This spatial organization reflects the Darwinian evolution of subclones within varied tumor microenvironments, where hypoxic and angiogenic subregions create niches for the emergence of metastatic variants. The clinical implementation of habitat-based risk stratification could potentially revolutionize neuro-oncological surveillance in NSCLC, moving from population-based schedules toward individualized risk-adapted protocols.

## 5. Limitations

Our study also has several limitations. First, the retrospective nature, single center design and a relatively small sample size may induce selection bias, which could constrain the generalizability of the findings. A prospective, multicenter study is needed to further confirm the predictive efficiency. Second, the absence of an external validation poses challenges in practical application of the proposed model. Third, although the K-means clustering algorithm is practical, it struggles to capture the irregular morphology of tumors due to its spherical assumption, its reliance on a pre-set K value can distort tumor heterogeneity, and its ‘hard clustering’ characteristic means it cannot characterize the biological transition zones between subregions. Additionally, to improve the biological interpretability of the model’s predictions, we intend to incorporate association analyses involving genetic and molecular markers in future studies.

## 6. Conclusions

We developed a novel habitat-based model analyzing primary lung tumor characteristics to predict BM in patients with NSCLC. This model demonstrated superior predictive performance compared with conventional radiomics approaches, as evidenced by higher AUC values (0.819 vs. 0.728) and improved sensitivity. While these findings require prospective validation in larger multicenter cohorts, the model demonstrated clinical potential in terms of its ability to stratify patients according to BM risk with greater accuracy. Accurate stratification can inform personalized surveillance protocols and guide therapeutic decision-making, ultimately supporting early intervention strategies for high-risk patients while sparing low-risk individuals from unnecessary prophylactic treatments.

## Figures and Tables

**Table 1:** Characteristics of NSCLC patients in training and validation groups.

Characteristics	Training Cohort (*n* = 138)	Validation Cohort (*n* = 57)	Statistic	*p*-Value
Age	65.76 ± 9.15	65.95 ± 8.12	0.134	0.894
Gender (male%)	79 (57.2%)	36 (63.2%)	0.583	0.445
CEA (0–3 ng/mL)	5.61 (2.90–24.95)	10.70 (4.08–62.13)	−1.491	0.136
CA125 (0–35 U/mL)	19.80 (12.07–78.41)	23.50 (20.20–79.31)	−1.053	0.292
CA72-4 (0–6.9 IU/mL)	4.21 (1.56–11.14)	5.39 (2.73–11.14)	−1.480	0.139
NSE (0–16.3 ng/mL)	12.33 (10.57–15.71)	14.38(12.06–18.99)	−2.196	0.028
SCC (0–1.5 ng/mL)	1.00 (0.70–2.06)	0.90 (0.50–1.20)	−0.845	0.396
ProGRP (0–70 pg/mL)	36.57 (27.73–42.95)	34.46 (26.30–41.99)	−0.711	0.477
Cyfra21-1 (0–2.08 ng/mL)	2.53 (1.66–6.51)	3.79 (1.94–6.51)	−1.572	0.116
Smoking history	63 (45.7%)	29 (50.9%)	0.442	0.506
Tumor type			4.153	0.042
Adenocarcinoma	124 (89.9%)	45 (78.9%)		
Squamous Cell Carcinoma	14 (10.1%)	12 (21.1%)		
Tumor classification			1.304	0.521
Peripherally located	120 (87.0%)	47 (82.5%)		
Centrally located	18 (13.7%)	11 (17.5%)		
T stage			9.942	0.269
I	26 (18.8%)	12(21.1%)		
II	42 (30.4%)	19 (33.3%)		
III	32 (23.2%)	14 (24.60%)		
IV	38 (27.5%)	12 (21.1%)		
N stage			4.557	0.207
N0	21 (36.8%)	34 (24.6%)		
N1	13 (22.8%)	27 (19.6%)		
N2	16 (28.1%)	48 (34.8%)		
N3	7 (12.3%)	29 (21.0%)		
M stage			11.006	0.09
M0	64 (46.4%)	30 (52.6%)		
M1 ^#^	52 (53.6%)	17 (47.4%)		
Gene mutation	89 (64.5%)	38 (66.7%)	0.869	0.453
EGFR	65 (47.1%)	29 (50.9%)	0.640	0.373
ALK	6 (4.3%)	2 (3.5%)	1.00	0.571
KRAS	13 (9.4%)	7 (12.3%)	0.606	0.358
TP53	15 (10.9%)	4 (7.0%)	0.596	0.296
Other	3 (2.2%)	NA		

NSCLC: non-small cell lung cancer, CEA: carcinoembryonic Antigen, CA125: carbohydrate antigen 125, CA72-4: carbohydrate antigen 72-4, NSE: neuro-specific enolase, SCC: squamous cell carcinoma antigen, ProGRP: pro-gastrin releasing peptide, Cyfra21-1: cytokeratin 19 fragment, EGFR: epidermal growth factor receptor, ALK: anaplastic Lymphoma kinase, KRAS: kirsten rat sarcoma viral oncogene, TP53: tumor protein p53. ^#^ single/multiple distant sites, organs, or peritoneal metastases detected by imaging or pathology.

**Table 2:** The performance of two models for BM prediction in NSCLC.

Model	Training Cohort (*n* = 138)			Validation Cohort (*n* = 57)		
	AUC (95% CI)	Sensitivity	Specificity	AUC (95% CI)	Sensitivity	Specificity
Model_clin_	0.639 (0.543–0.731)	0.854	0.313	0.613 (0.457–0.769)	0.789	0.364
Model_VOI_	0.728 (0.645–0.812)	0.788	0.593	0.690 (0.542–0.839)	0.571	0.778
Model_Sub_	0.819 (0.744–0.894)	0.788	0.791	0.772 (0.641–0.904)	0.810	0.750

BM: brain metastasis, NSCLC: non-small cell lung cancer, AUC: area under the curve, and CI: confidence interval.

## Data Availability

The clinical datasets and segmentations of whole-tumor regions of interest supporting the findings of this study are openly available in [Mendeley Data] at https://data.mendeley.com/datasets/kpfrhzxyhg/1, accessed on 2 November 2025). However, anonymized CT data may be available from the corresponding author on reasonable request.
